# Mortality data from omission of early thromboprophylaxis in critically ill patients highlights the importance of an individualised diagnosis-related approach

**DOI:** 10.1186/s12959-023-00499-y

**Published:** 2023-05-23

**Authors:** Berhe W. Sahle, David Pilcher, Karlheinz Peter, James D. McFadyen, Edward Litton, Tracey Bucknall

**Affiliations:** 1 School of Nursing and Midwifery, Faculty of Health, Deakin University, Melbourne, VIC Australia; 2 Centre for Quality and Patient Safety Research, Alfred Health Partnership, Institute for Health Transformation, Melbourne, VIC Australia; 3 Department of Intensive Care, Alfred Hospital, Melbourne, VIC Australia; 4 School of Public Health and Preventive Medicine, Monash University, Melbourne, VIC Australia; 5 Australian and New Zealand Intensive Care Society Centre for Outcome and Resource Evaluation, Melbourne, VIC Australia; 6 Atherothrombosis and Vascular Biology, Baker Heart and Diabetes Institute, Melbourne, VIC Australia; 7 Department of Medicine, Central Clinical School, Monash University, Melbourne, VIC Australia; 8 Baker Department of Cardiometabolic Health, University of Melbourne, Melbourne, VIC Australia; 9 Department of Cardiology, The Alfred Hospital, Melbourne, VIC Australia; 10 Department of Clinical Hematology, The Alfred Hospital, Melbourne, VIC Australia; 11 Fiona Stanley Hospital, Perth, WA Australia; 12 The University of Western Australia, Perth, WA Australia

**Keywords:** Venous thromboembolism; VTE, Thromboprophylaxis, Intensive care unit, Critically ill, Mortality

## Abstract

**Background:**

Venous thromboembolism (VTE) prophylaxis is effective in reducing VTE events, however, its impact on mortality is unclear. We examined the association between omission of VTE prophylaxis within the first 24 h after intensive care unit (ICU) admission and hospital mortality.

**Methods:**

Retrospective analysis of prospectively collected data from the Australian New Zealand Intensive Care Society Adult Patient Database. Data were obtained for adult admissions between 2009 and 2020. Mixed effects logistic regression models were used to evaluate the association between omission of early VTE prophylaxis and hospital mortality.

**Results:**

Of the 1,465,020 ICU admissions, 107,486 (7.3%) did not receive any form of VTE prophylaxis within the first 24 h after ICU admission without documented contraindication. Omission of early VTE prophylaxis was independently associated with 35% increased odds of in-hospital mortality (odds ratios (OR): 1.35; 95% CI: 1.31–1.41). The associations between omission of early VTE prophylaxis and mortality varied by admission diagnosis. In patients diagnosed with stroke (OR: 1.26, 95% CI: 1.05–1.52), cardiac arrest (OR: 1.85, 95% CI: 1.65–2.07) or intracerebral haemorrhage (OR: 1.48, 95% CI: 1.19–1.84), omission of VTE prophylaxis was associated with increased risk of mortality, but not in patients diagnosed with subarachnoid haemorrhage or head injury.

**Conclusions:**

Omission of VTE prophylaxis within the first 24 h after ICU admission was independently associated with increased risk of mortality that varied by admission diagnosis. Consideration of early thromboprophylaxis may be required for patients with stroke, cardiac arrest and intracerebral haemorrhage but not in those with subarachnoid haemorrhage or head injury. The findings highlight the importance of individualised diagnosis-related thromboprophylaxis benefit-harm assessments.

**Supplementary Information:**

The online version contains supplementary material available at 10.1186/s12959-023-00499-y.

## Background

Venous thromboembolism (VTE) is the leading cause of preventable death in hospitals, a leading contributor to increased length of stay, and is the number one patient safety priority [1, 2]. Globally, there are about 10 million cases of VTE every year, and around 30% of patients with VTE experience recurrence within 10 years [3]. The long-term mortality rate in untreated VTE patients ranges from 12 to 50% [4]. In addition to the impact of VTE on morbidity and mortality, VTE imposes a significant economic burden on patients, their families and the healthcare system [5]. In Australia, more than 17,000 people develop VTE each year, which costs patients and the health system $1.72 billion annually [6]. Although VTE is common in hospital patients, it is more prevalent in critically ill patients due to specific ICU risk factors of VTE, including sedation, immobilization, vasopressors or central venous catheterisation [7].

The use of appropriate VTE prophylaxis reduces the risk of VTE by 50–80% but may increase the risk of bleeding [1, 5, 8]. Current guidelines recommend that all hospital patients should be assessed for VTE risk and most patients should receive appropriate VTE prophylaxis within the first 24 h after admission [6, 9]. However, a significant proportion of patients at risk of having VTE do not receive VTE prophylaxis as recommended in guidelines [5, 10]. There is also considerable variation in VTE prophylaxis use and incident VTE between hospitals in Australia [11, 12] and globally [10], independent of differences in the proportion of patients at risk for VTE. The variation and underuse of VTE prophylaxis have been mainly attributed to uncertainty among clinicians as to how the benefits and risks of anticoagulants weigh up in patients with different risk profiles [13].

The association between delay or omission of VTE prophylaxis and increased risk of VTE is well established [14, 15]. However, current evidence on the association between delay or omission of VTE prophylaxis and mortality is not clear. Some studies [14, 16] have found an association between delay or omission of VTE prophylaxis and increased risk of mortality, whereas other studies reported that there is no association between delay or mission of early VTE prophylaxis and mortality [15, 17, 18]. In a multi-centre registry study of 175,665 critically ill adults, omission of VTE prophylaxis in the first 24 h after ICU admission was associated with 1.22-fold increase in hospital mortality, ranging from 1.07 to 1.88 fold depending on admission diagnosis and pre-existing chronic conditions [16]. However, meta-analyses studies [15, 18] and a large registry study [14] found no statistically significant difference in mortality between early versus late VTE prophylaxis. The extent of mortality attributable to omission of early thromboprophylaxis, and whether adverse outcomes attributable to omission of early thromboprophylaxis differ according to patients’ risk profiles remains unclear. Overall, current guidelines do not provide admission diagnosis specific recommendations for VTE prophylaxis use. The lack of standardised approaches to assess bleeding risk, including in cardiac arrest, intracerebral hemorrhage and stroke patients could influence the decision whether or not to prescribe pharmacological prophylaxis.

The lack of clear evidence between omission of early VTE prophylaxis and mortality has, in part, contributed to weak guideline recommendations and heterogeneity of practice. Larger sampled studies of contemporaneous patients are needed for identifying VTE prophylaxis strategies associated with lower risk of death among critically ill and clinically diverse patient groups. In this study, we investigated the association between omission of VTE prophylaxis and in-hospital mortality in a very large cohort of patients admitted to Intensive Care Units in Australia and New Zealand.

## Methods

### Data sources and participants

This is a retrospective analysis of data from the Australian and New Zealand Intensive Care Society (ANZICS) Adult Patient Database (APD) run by the ANZICS Centre for Outcome and Resource Evaluation (CORE). The ANZICS APD contains over 3 million patient episodes collected from 221 ICUs, representing about 90% of Australia and New Zealand ICU admissions. Additional details regarding the design and methods of the ANZICS APD study are described elsewhere [19].

The outcome of this study was hospital mortality, which was reported by the contributing ICUs. ICUs reported administration of VTE prophylaxis status within the first 24 h of ICU admission using a standard precoded response: “Yes,” “No,” “Contraindicated,” or “Not indicated.” Early VTE prophylaxis was defined as receiving one or more of the following methods of VTE prophylaxis, including unfractionated heparin, low molecular-weight heparin, pneumatic compression devices, compression stockings, or inferior vena cava filter, within the first 24 h after ICU admission [16, 20]. Omission of early VTE prophylaxis was defined as not receiving any method of VTE prophylaxis within the first 24 h after ICU admission without obvious reasons or contraindications to pharmacologic or mechanical VTE prophylaxis. Patients were considered as “not indicated” for early VTE prophylaxis if they did not receive any form of VTE prophylaxis within the first 24 h after ICU admission because they were assessed to have a very low risk of VTE events by their attending clinicians. VTE prophylaxis was considered contraindicated if patient was at risk of bleeding and had physical injuries to their lower extremities, unless an inferior vena cava filter was inserted within the first 24 h after ICU admission [16].

The analyses were adjusted for risk of hospital mortality estimated using the Australian and New Zealand Risk of Death (ANZROD) model. The ANZROD model has excellent discrimination and good calibration and provides better adjustment for case mix variation [21]. ANZROD is derived from patient and clinical characteristics, including the Acute Physiology and Chronic Health Evaluation (APACHE) III, ICU admission source, admission diagnoses, Acute Physiology score (APS), APACHE III chronic health score, treatment limitation, and ventilation [22].

### Data analysis

Demographic and clinical characteristics are presented as means (with SDs) for continuous variables and percentages for categorical variables. We used mixed effects logistic regression modeling (accounting for the random effects of the contributing ICUs) to identify the association between omission of early VTE prophylaxis and hospital mortality overall and stratified according to admission diagnosis and pre-existing chronic conditions. Temporal changes in the association between omission of early VTE prophylaxis and mortality were assessed by including an interaction term between calendar year and VTE prophylaxis in the fully adjusted mixed effects logistic regression. P < 0.05 was considered significant in 2-sided tests. The proportion of missing data was negligible, and therefore, imputation methods were not necessary. Analyses were carried out using Stata version 16 (StataCorp, College Station, TX, USA).

The study was approved by the Alfred Health Human Research Ethics Committee (Project No: 276/21). The ANZICS CORE participating ICUs contribute de-identified data. Contributing ICUs allow subsequent data use as appropriate, understanding procedures and in compliance with the ANZICS CORE terms of reference and with a waiver of the need for informed consent.

## Results

### Study subjects

**Fig. 1:**
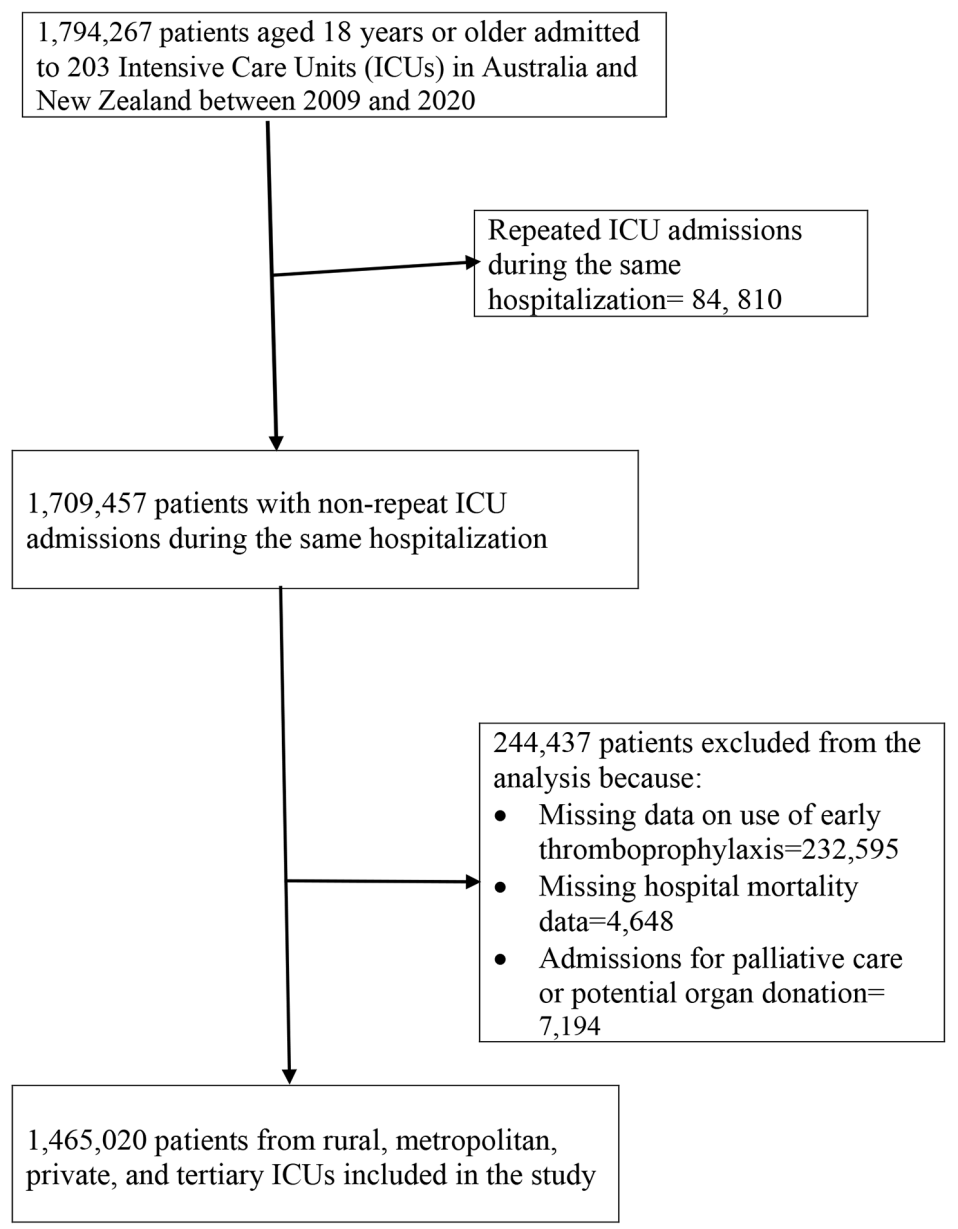
Inclusion and exclusion criteria and number of patients

A total of 1,465,020 index admissions of patients aged 18 years or older (median age 65.3 ± 17.5 y, 43.3% females) admitted to 203 ICUs in Australia and New Zealand met the inclusion criteria for the analysis (Fig. 1). Patients with missing data on early thromboprophylaxis (n = 232,595) or hospital mortality (n = 4, 648), and admitted for palliative care or potential organ donation (n = 7,194) were excluded. Patient characteristics by early thromboprophylaxis status are shown in Table 1.

**Table 1:** Characteristics of the patients included in the analysis (n = 1,465,020)

Characteristic	Early thromboprophylaxis within 24 h of ICU admission			
	Yes 1,260,785 (86.1%)	No 107,486 (7.3%)	Contraindicated 55,025 (3.8%)	Not indicated 41,724 (2.9%)
Age (years), median (IQR)	65.5 (51.6–75.4)	63.7 (47.7–75.0)	66.1 (51.3–76.9)	62.8 (47.0–74.0)
Male	714,183 (56.7)	59,990 (55.8)	32, 152 (58.4)	24,725 (59.3)
Female	546,133 (43.3)	47,469 (44.2)	22, 853 (41.6)	16,989 (40.7)
**Admission diagnosis**				
Head trauma with or without multi trauma	12,438 (79.4)	1,471 (9.4)	1,425 (9.1)	325 (2.1)
Cardiac arrest	25,350 (85.6)	2,506 (8.5)	927 (3.1)	819 (2.8)
Intracerebral haemorrhage	4,943 (66.1)	873 (11.7)	1,502 (20.1)	158 (2.1)
Subarachnoid haemorrhage	5,211 (78.9)	525 (7.9)	789 (11.9)	83 (1.3)
Stroke	8,814 (75.7)	1,153 (9.9)	1,388 (11.9)	290 (2.5)
Cardiovascular disease	304,248 (24.0)	25,201 (23.3)	8,819 (15.9)	15,968 (38.0)
Respiratory disease	193,278 (15.3)	16,387 (15.2)	4,027 (8.2)	6,102 (14.5)
Sepsis	89,523 (7.1)	7,882 (7.3)	4,700 (8.4)	2,239 (5.3)
**Pre-existing chronic conditions**				
Chronic respiratory disease	93,399 (7.4)	7,521 (7.0)	3,549 (6.4)	2,671 (6.4)
Chronic cardiovascular disease	112,346 (8.9)	9,313 (8.7)	6,547 (11.9)	3,391 (8.1)
Chronic renal failure	41,055 (3.3)	3,963 (3.7)	2,570 (4.7)	1,558 (3.7)
Chronic liver disease	18,523 (1.5)	2,407 (2.2)	3,531 (6.4)	647 (1.5)
Immunosuppressive disease	26,801 (2.1)	2,050 (1.9)	1,629 (3.0)	510 (1.2)
Metastatic cancer	50, 100 (4.0)	3,130 (2.9)	2,085 (3.8)	889 (2.1)
APACHE III score, median (IQR), %	48.0 (35.0–64.0)	47.0 (33.0–66.0)	54.0 (38.0–75.0)	46.0 (32.0–62.0)
APACHE III predicted mortality, mean (SD),%	12.5 (18.6)	14.4 (21.7)	21.2 (25.4)	11.8 (20.0)
ANZROD (%), mean (SD)	7.4 (15.5)	9.4 (19.1)	15.2 (23.6)	7.5 (17.1)
Length of hospital stay, median (IQR), d	8.1 (4.5–14.7)	6.6 (2.8–12.9)	7.8 (3.7–15.9)	6.5 (2.8–11.5)
Hospital mortality	89,891 (7.1)	11,285 (10.5)	8,723 (15.8)	3,376 (8.1)

APACHE- Acute Physiology and Chronic Health Evaluation; ANZROD- Australian and New Zealand Risk of Death; ICU: Intensive Care Unit, SD- standard deviation, d- days

## Early VTE prophylaxis and mortality

In total, 1,260,785 (86.1%) of ICU patients received VTE prophylaxis within the first 24 h after ICU admission. Omission of VTE prophylaxis without documented contraindication occurred in 107,486 (7.3%) ICU patients. VTE prophylaxis was contraindicated in 55,025 (3.8%) patients, and not indicated in 41,724 patients (2.9%). The hospital mortality rate in patients with omission of early thromboprophylaxis was higher compared to patients who received early VTE prophylaxis (10.5% vs. 7.1%) despite the former patients had longer hospital lengths of stay and severity of illness scores (Table 1).

## Association of omission of early VTE prophylaxis with mortality

In the analysis adjusted for ANZROD, omission of early VTE prophylaxis (odds ratios (OR): 1.35; 95% CI: 1.31–1.40), or contraindications (OR: 1.35; 95% CI: 1.31–1.40) but not no indication (1.22; 95% CI: 0.94–1.14) to VTE prophylaxis was associated with significantly increased odds of hospital death. The increased mortality risk of mortality associated to omission of early VTE prophylaxis was consistent when stratified according to pre-existing chronic conditions (Table 2). After adjusting for ANZROD, the risk of mortality attributable to omission of early VTE prophylaxis varied according to admission diagnosis. In the analyses stratified by admission diagnosis, omission of VTE prophylaxis was associated with 23–85% statistically significant increased odds of mortality, except in patients diagnosed with head trauma (with or without multi trauma) (OR: 1.23; 95% CI: 0.97–1.56) or subarachnoid haemorrhage (OR: 1.09; 95% CI: 0.79–1.50) (Table 3). There was no statistically significant interaction (P-for interaction effect > 0.05) between ICU admission year and VTE prophylaxis on hospital mortality.

**Table 2:** Association between early thromboprophylaxis and mortality by pre-existing medical conditions

Pre-existing medical conditions	Odds ratios (95% CI)	*P-value*
Chronic respiratory disease (n = 106,833)		
VTE prophylaxis		
Yes	Ref	
No	1.38 (1.27–1.50)	*< 0.001*
Contraindicated	1.10 (0.99–1.23)	0.069
Not indicated	1.31 (1.15–1.51)	*< 0.001*
Chronic cardiovascular disease (n = 131,038)		
VTE prophylaxis		
Yes	Ref	
No	1.46 (1.34–1.57)	< 0.001
Contraindicated	1.28 (1.18–1.40)	< 0.001
Not indicated	1.24 (1.09–1.41)	0.001
Chronic renal failure (n = 48,970)		
VTE prophylaxis		
Yes	Ref	
No	1.34 (1.20–1.49)	< 0.001
Contraindicated	1.32 (1.17–1.49)	< 0.001
Not indicated	1.32 (1.10–1.57)	0.026
Chronic liver disease (n = 25,058)		
VTE prophylaxis		
Yes	Ref	
No	1.27 (1.11–1.45)	< 0.001
Contraindicated	1.23 (1.11–1.37)	< 0.001
Not indicated	1.30 (1.03–1.63)	0.026
Metastatic cancer (n = 56,101)		
VTE prophylaxis		
Yes	Ref	
No	1.37 (1.21–1.54)	< 0.001
Contraindicated	1.34 (1.17–1.54)	< 0.001
Not indicated	1.29 (1.03–1.63)	0.029

Adjusted for Australian and New Zealand Risk of Death (ANZROD). ANZROD is derived from patient and clinical characteristics, including the Acute Physiology and Chronic Health Evaluation (APACHE) III, ICU admission source, admission diagnoses, Acute Physiology score (APS), APACHE III chronic health score, treatment limitation, and ventilation status. VTE-Venous thromboembolism

**Table 3:** Association between early thromboprophylaxis and mortality by admission diagnosis

Characteristic	Odds ratios (95% CI)	*P-value*
**Overall**		
Thromboprophylaxis status		
Yes	Ref	
No	1.35 (1.31–1.40)	< 0.001
Contraindicated	1.35 (1.31–1.40)	< 0.001
Not indicated	1.22 (0.94–1.12)	0.522
**Admission diagnosis**		
Head trauma (with or without multi trauma) (n = 15,618)		
Yes	Ref	
No	1.23 (0.97–1.56)	0.090
Contraindicated	1.20 (0.95–1.51)	0.115
Not indicated	1.71 (1.08–2.69)	0.020
Cardiac arrest (n = 29,498)		
VTE prophylaxis		
Yes	Ref	
No	1.85 (1.65–2.07)	*< 0.001*
Contraindicated	1.37 (1.16–1.62)	*< 0.001*
Not indicated	1.29 (1.91–2.75)	*< 0.001*
Intracerebral haemorrhage (n = 7,448)		
Yes	Ref	
No	1.48 (1.19–1.84)	*< 0.001*
Contraindicated	1.14 (0.96–1.37)	0.136
Not indicated	2.41 (1.52–3.82)	*< 0.001*
Subarachnoid haemorrhage (n = 6,598)		
Yes	Ref	
No	1.09 (0.79–1.50)	0.582
Contraindicated	0.97 (0.74–1.26)	0.824
Not indicated	1.38 (0.73–2.59)	0.316
Stroke (n = 11,615)		
Yes	Ref	
No	1.26 (1.05–1.52)	0.014
Contraindicated	1.07 (0.90–1.27)	0.457
Not indicated	1.16 (0.82–1.63)	0.392

Adjusted for Australian and New Zealand Risk of Death (ANZROD). ANZROD is derived from patient and clinical characteristics, including the Acute Physiology and Chronic Health Evaluation (APACHE) III, ICU admission source, admission diagnoses, Acute Physiology score (APS), APACHE III chronic health score, treatment limitation, and ventilation status. VTE-Venous thromboembolism

## Discussion

In this multi-centre registry of more than 1.4 million critically ill patients, omission of early VTE phylaxis without obvious reasons or contraindications to VTE prophylaxis was independently associated with increased risk of mortality. The association between omission of early VTE prophylaxis varied substantially by admission diagnosis. In stroke, cardiac arrest, and intracerebral haemorrhage, omission of early thromboprophylaxis was associated with increased risk of mortality but not in those with subarachnoid haemorrhage or head injury.

There could be several possible reasons for the higher risk of mortality in patients who do not receive early VTE prophylaxis. First, the higher risk of death associated with omission of early VTE prophylaxis could be attributed to the high incidence of VTE and complications in patients at risk of VTE who did not receive VTE prophylaxis [4, 14]. Without appropriate VTE prophylaxis, the overall VTE incidence in medical and general surgery hospitalized patients ranges from 10 to 40%, while it ranges up to 40–60% in major orthopaedic surgery [23], and one in 10 patients who developed hospital-acquired VTE die in hospital [12]. Second, VTE prophylaxis is a key indicator of quality of care for hospitalized patients, and omission of early VTE prophylaxis without obvious reasons or contraindications to pharmacologic or mechanical thromboprophylaxis may reflect gaps in the overall quality of care, including physician awareness, compliance with guidelines for VTE prophylaxis [24, 25]. Previous research has suggested that improvement in prescription of risk appropriate VTE prophylaxis, reduces symptomatic VTE and preventable harm from VTE without increasing major bleeding [26].

The inconsistencies between studies in the association between VTE prophylaxis and mortality could be, in part, attributed to case mix variation, including comorbidities and admission diagnosis which affect the risk of mortality and the decision whether and the type of VTE prophylaxis prescribed [14, 17, 27]. First, the effectiveness of individual VTE prophylaxis in reducing VTE events is established, however, the balance of effectiveness and safety depends on the patient’s risk profiles [28]. Patients have different risk profiles, such as VTE risk factors, risk of bleeding, admission diagnosis, and some groups of patients may not benefit from VTE prophylaxis if their baseline risk of VTE is low or the associated risk of major bleeding is high [13, 29]. Second, differences in the association between omission of VTE prophylaxis and mortality between studies could be due to difference in prophylactic agent selection, dose, duration and timing of initiation of VTE prophylaxis [13, 14]. Third, omission of VTE prophylaxis could be due to other reasons, including patient preference, prescribing omission, medication errors or perceived unnecessary which could lead to a varying impact of omission of VTE on mortality [30]. For example, in cardiac arrest, intracerebral hemorrhage and stroke patients, there are no standardised ways to assess bleeding risk, which in turn plays a key role on determining whether pharmacological prophylaxis is given. Our analyses were adjusted for several risk factors that might influence use of VTE prophylaxis, including severity of illness, chronic health conditions, and admission diagnosis.

There is limited evidence regarding the mortality benefit of VTE prophylaxis in patients diagnosed with neurological conditions [17] and traumatic brain injury (TBI) [31], which could be among the reasons why omission of VTE prophylaxis was not associated with mortality in patients admitted due to subarachnoid haemorrhage or head trauma. A recent meta-analysis of patients undergoing neurosurgical interventions did not find evidence of the association between pharmacologic VTE prophylaxis and mortality [17]. Analysis of Victorian State Trauma Registry from July 2017 to June 2018, showed that in older patients with major trauma anticoagulant use was associated with higher odds of in-hospital mortality [32]. Exiting evidence suggest that patients diagnosed with intracerebral haemorrhage with indication for thromboprophylaxis benefit from pharmacological prophylaxis without significantly increasing bleeding complications [33, 34], whereas in initiation of mechanical prophylaxis is recommended in Subarachnoid haemorrhage patients [34]. The difference in VTE prophylaxis related mortality between intracerebral and subarachnoid haemorrhage patients could be because of variations in the location or cause of haemorrhage which influence disease management and outcomes. These could be among the reasons why the risk of mortality was not significantly higher in patients diagnosed with head injury or subarachnoid haemorrhage who missed out VTE prophylaxis. However, it is not clear from this study why there appear to be differences between the brain injury diagnoses groups.

Our findings have implications for improving VTE prevention and care, since they demonstrate the importance of individualising VTE prophylaxis decision making. Evidence-based guidelines acknowledge that VTE prophylaxis methods differ in their balance of benefits and harms depending on the patient’s risk profile and recommends balancing VTE risk and bleeding risk [6]. However, there are no tools for objective assessment of the benefit-harm balance of VTE prophylaxis, and VTE prophylaxis decisions are made based on clinician’s own judgment, often guided by anecdote or experience [27]. The lack of understanding how to individualize VTE prophylaxis decision making has been identified as a critical gap in VTE prevention practices [28].

Our study has some limitations. The variables which are potentially important for understanding VTE prophylaxis use, including VTE and bleeding risk assessment, reasons for not receiving VTE prophylaxis and type, dose and duration of VTE prophylaxis were not collected, therefore were not taken into account in the analyses. Contextual factors that influence VTE prophylaxis decision making such as patient preference, awareness of VTE prophylaxis, and local VTE prophylaxis guidelines were not collected. Furthermore, sometimes clinicians or data collectors may not differentiate between ‘contraindicated’ and ‘not indicated’, which is another potential limitation. Finally, data on treatments given in the ICU, and how many patients were already on anticoagulants when they come to ICU were not collected, therefore were not taken into account in the analyses.

## Conclusions

In this multi-centre registry study including more than 1.4 million ICU admissions, omission of VTE prophylaxis within the first 24 h after ICU admission without obvious reasons or contraindications to VTE prophylaxis was independently associated with increased risk of in-hospital mortality. The risk of in-hospital mortality attributable to omission of VTE prophylaxis varied substantially by admission diagnosis. In stroke, cardiac arrest, intracerebral haemorrhage, thromboprophylaxis might be indicated but not in those with subarachnoid haemorrhage or head injury. The findings highlight the importance of individualised diagnosis-related thromboprophylaxis practices.

## Electronic supplementary material

Below is the link to the electronic supplementary material.

Supplementary Material 1

## Data Availability

The datasets used in the current study are not publicly available, but are available from the corresponding author on reasonable request.

## Abbreviations

ANZICS: Australian and New Zealand Intensive Care Society
ANZROD: Australian and New Zealand Risk of Death
APACHE: Acute Physiology and Chronic Health Evaluation
APD: Adult Patient Database
CORE: Centre for Outcome and Resource Evaluation
ICU: intensive care unit; VTE:Venous thromboembolism
OR: Odds Ratio
