# Supplementary material for: Mortality data from omission of early thromboprophylaxis in critically ill patients highlights the importance of an individualised diagnosis-related approach
Source: Thromb J. 2023 May 23;21:59. doi: 10.1186/s12959-023-00499-y (PMC10207702; doi:10.1186/s12959-023-00499-y)
Supplement: Supplementary file 1 — Supplementary Material 1 [file 12959_2023_499_MOESM1_ESM.docx]

**Supplementary Table 1: List of participating hospital in the study**

Albury Base Hospital ICU; Alfred Hospital ICU; Alice Springs Hospital ICU; Allamanda Private Hospital ICU; Angliss Hospital ICU; Armadale Health Service ICU; Ashford Community Hospital ICU; Auckland City Hospital CV ICU; Auckland City Hospital DCCM; Austin Hospital ICU; Ballarat Health Services ICU; Bankstown-Lidcombe Hospital ICU; Bathurst Base Hospital ICU; Bendigo Health Care Group ICU; Blacktown Hospital ICU; Box Hill Hospital ICU; Brisbane Private Hospital ICU; Brisbane Waters Private Hospital ICU; Buderim Private Hospital ICU; Bunbury Regional Hospital ICU; Bundaberg Base Hospital ICU; Caboolture Hospital ICU; Cabrini Hospital ICU; Cairns Hospital ICU; Calvary Bruce Private Hospital HDU; Calvary Hospital (Canberra) ICU; Calvary Hospital (Lenah Valley) ICU; Calvary John James Hospital ICU; Calvary Mater Newcastle ICU; Calvary North Adelaide Hospital ICU; Calvary Wakefield Hospital (Adelaide) ICU; Campbelltown Hospital ICU; Canberra Hospital ICU; Casey Hospital ICU; Central Gippsland Health Service ICU; Christchurch Hospital ICU; Coffs Harbour Health Campus ICU; Concord Hospital (Sydney) ICU; Dandenong Hospital ICU; Dubbo Base Hospital ICU; Dunedin Hospital ICU; Epworth Eastern Private Hospital ICU; Epworth Freemasons Hospital ICU; Epworth Geelong ICU; Epworth Hospital (Richmond) ICU; Fairfield Hospital ICU; Figtree Private Hospital ICU; Fiona Stanley Hospital ICU; Flinders Medical Centre ICU; Flinders Private Hospital ICU; Footscray Hospital ICU; Frankston Hospital ICU; Fremantle Hospital ICU; Gold Coast Private Hospital ICU; Gold Coast University Hospital ICU; Gosford Hospital ICU; Gosford Private Hospital ICU; Goulburn Base Hospital ICU; Goulburn Valley Health ICU; Grafton Base Hospital ICU; Greenslopes Private Hospital ICU; Griffith Base Hospital ICU; Hawkes Bay Hospital ICU; Hervey Bay Hospital ICU; Hollywood Private Hospital ICU; Holmesglen Private Hospital ICU; Holy Spirit Northside Hospital ICU; Hornsby Ku-ring-gai Hospital ICU; Hurstville Private Hospital ICU; Hutt Hospital ICU; Ipswich Hospital ICU; John Fawkner Hospital ICU; John Flynn Private Hospital ICU; John Hunter Hospital ICU; Joondalup Health Campus ICU; Kareena Private Hospital ICU; Knox Private Hospital ICU; Latrobe Regional Hospital ICU; Launceston General Hospital ICU; Lingard Private Hospital ICU; Lismore Base Hospital ICU; Liverpool Hospital ICU; Logan Hospital ICU; Lyell McEwin Hospital ICU; Mackay Base Hospital ICU; Macquarie University Private Hospital ICU; Maitland Hospital HDU/CCU; Maitland Private Hospital; Manly Hospital & Community Health ICU; Manning Rural Referral Hospital ICU; Maroondah Hospital ICU; Mater Adults Hospital (Brisbane) ICU; Mater Health Services North Queensland ICU; Mater Private Hospital (Brisbane) ICU; Mater Private Hospital (Sydney) ICU; Melbourne Private Hospital ICU; Middlemore Hospital ICU; Mildura Base Hospital ICU; Modbury Public Hospital ICU; Monash Medical Centre-Clayton Campus ICU; Mount Hospital ICU; Mount Isa Hospital ICU; Nambour General Hospital ICU; National Capital Private Hospital ICU; Nelson Hospital ICU; Nepean Hospital ICU; Nepean Private Hospital ICU; Newcastle Private Hospital ICU; Noosa Hospital ICU; North Shore Hospital ICU; North Shore Private Hospital ICU; North West Regional Hospital (Burnie) ICU; Northeast Health Wangaratta ICU; Northern Beaches Hospital; Norwest Private Hospital ICU; Orange Base Hospital ICU; Peninsula Private Hospital ICU; Peter MacCallum Cancer Institute ICU; Pindara Private Hospital ICU; Port Macquarie Base Hospital ICU; Prince of Wales Hospital (Sydney) ICU; Prince of Wales Private Hospital (Sydney) ICU; Princess Alexandra Hospital ICU; Queen Elizabeth II Jubilee Hospital ICU; Redcliffe Hospital ICU; Repatriation General Hospital (Adelaide) ICU; Robina Hospital ICU; Rockhampton Hospital ICU; Rockingham General Hospital ICU; Rotorua Hospital ICU; Royal Adelaide Hospital ICU; Royal Brisbane and Women's Hospital ICU; Royal Darwin Hospital ICU; Royal Hobart Hospital ICU; Royal Melbourne Hospital ICU; Royal North Shore Hospital ICU; Royal Perth Hospital ICU; Royal Prince Alfred Hospital ICU; Ryde Hospital & Community Health Services ICU; Shoalhaven Hospital ICU; Sir Charles Gairdner Hospital ICU; South West Healthcare (Warrnambool) ICU; Southern Cross Hospital (Hamilton) ICU; Southern Cross Hospital (Wellington) ICU; St Andrew's Hospital (Adelaide) ICU; St Andrew's Hospital Toowoomba ICU; St Andrew's Private Hospital (Ipswich) ICU; St Andrew's War Memorial Hospital ICU; St George Hospital (Sydney) CICU; St George Hospital (Sydney) ICU; St George Hospital (Sydney) ICU2; St George Private Hospital (Sydney) ICU; St John of God (Berwick) ICU; St John Of God Health Care (Subiaco) ICU; St John Of God Hospital (Ballarat) ICU; St John of God Hospital (Bendigo) ICU; St John Of God Hospital (Geelong) ICU; St John Of God Hospital (Murdoch) ICU; St John of God Midland Public & Private ICU; St Vincent's Hospital (Melbourne) ICU; St Vincent's Hospital (Sydney) ICU; St Vincent's Hospital (Toowoomba) ICU; St Vincent's Private Hospital (Sydney) ICU; St Vincent's Private Hospital Fitzroy ICU; Sunnybank Hospital ICU; Sunshine Coast University Hospital ICU; Sunshine Coast University Private Hospital ICU; Sunshine Hospital ICU; Sutherland Hospital & Community Health Services ICU; Sydney Adventist Hospital ICU; Sydney Southwest Private Hospital ICU; Tamworth Base Hospital ICU; Taranaki Health ICU; Tauranga Hospital ICU; The Bays Hospital ICU; The Chris O’Brien Lifehouse ICU; The Memorial Hospital (Adelaide) ICU; The Northern Hospital ICU; The Prince Charles Hospital ICU; The Queen Elizabeth (Adelaide) ICU; The Townsville Hospital ICU; The Valley Private Hospital ICU; The Wesley Hospital ICU; Timaru Hospital ICU; Toowoomba Hospital ICU; Tweed Heads District Hospital ICU; University Hospital Geelong ICU; Wagga Wagga Base Hospital & District Health ICU; Waikato Hospital ICU; Warringal Private Hospital ICU; Werribee Mercy Hospital ICU; Western District Health Service (Hamilton) ICU; Western Hospital (SA) ICU; Western Private Hospital ICU; Westmead Hospital ICU; Westmead Private Hospital ICU; Whakatane Hospital ICU; Whangarei Area Hospital, Northland Health Ltd ICU; Wimmera Health Care Group (Horsham) ICU; Wollongong Hospital ICU; Wollongong Private Hospital ICU; Women's and Children's Hospital PICU; Wyong Hospital ICU
